# Reperfusion Therapy with Rapamycin Attenuates Myocardial Infarction through Activation of AKT and ERK

**DOI:** 10.1155/2017/4619720

**Published:** 2017-03-08

**Authors:** Scott M. Filippone, Arun Samidurai, Sean K. Roh, Chad K. Cain, Jun He, Fadi N. Salloum, Rakesh C. Kukreja, Anindita Das

**Affiliations:** Pauley Heart Center, Department of Internal Medicine, Division of Cardiology, Virginia Commonwealth University, Richmond, VA 23298, USA

## Abstract

Prompt coronary reperfusion is the gold standard for minimizing injury following acute myocardial infarction. Rapamycin, mammalian target of Rapamycin (mTOR) inhibitor, exerts preconditioning-like cardioprotective effects against ischemia/reperfusion (I/R) injury. We hypothesized that Rapamycin, given at the onset of reperfusion, reduces myocardial infarct size through modulation of mTOR complexes. Adult C57 male mice were subjected to 30 min of myocardial ischemia followed by reperfusion for 1 hour/24 hours. Rapamycin (0.25 mg/kg) or DMSO (7.5%) was injected intracardially at the onset of reperfusion. Post-I/R survival (87%) and cardiac function (fractional shortening, FS: 28.63 ± 3.01%) were improved in Rapamycin-treated mice compared to DMSO (survival: 63%, FS: 17.4 ± 2.6%). Rapamycin caused significant reduction in myocardial infarct size (IS: 26.2 ± 2.2%) and apoptosis (2.87 ± 0.64%) as compared to DMSO-treated mice (IS: 47.0 ± 2.3%; apoptosis: 7.39 ± 0.81%). Rapamycin induced phosphorylation of AKT S473 (target of mTORC2) but abolished ribosomal protein S6 phosphorylation (target of mTORC1) after I/R. Rapamycin induced phosphorylation of ERK1/2 but inhibited p38 phosphorylation. Infarct-limiting effect of Rapamycin was abolished with ERK inhibitor, PD98059. Rapamycin also attenuated Bax and increased Bcl-2/Bax ratio. These results suggest that reperfusion therapy with Rapamycin protects the heart against I/R injury by selective activation of mTORC2 and ERK with concurrent inhibition of mTORC1 and p38.

## 1. Introduction

Timely intervention at reperfusion is the current standard of care for the treatment of an acute myocardial infarction (AMI). However, the consequences of reperfusion injury still represent a major limitation to treatment. Therefore, developing novel approaches to minimize such injury is needed to further improve outcomes following an AMI.

The mammalian target of Rapamycin (mTOR) is a serine/threonine kinase that functions as a key regulator of cell growth, survival, and metabolism [1]. Identified as an intracellular sensor, mTOR detects nutrient and energy availability, as well as stressors affecting the cell [1]. Complex downstream signaling networks provide mTOR the ability to regulate autophagy, protein synthesis, cell polarity, and cytoskeletal organization [2–6]. For this reason, mTOR signaling has been studied extensively to elucidate its role in diabetes, aging, cancer, metabolic disorders, and cardiovascular diseases [2, 7–12]. mTOR is also an integral component for cardiovascular development and its targeted deletion in the heart has been shown to result in cardiomyopathy, heart failure, and death [5].

Effective metabolic control is established via the formation of two distinct mTOR multiprotein complexes: mTOR complex 1 (mTORC1) and mTOR complex 2 (mTORC2) [6, 7, 13]. These complexes are responsible for the regulation of different cellular processes and are defined by the adaptor proteins that bind to the central mTOR catalytic subunit. Common to both complexes are DEPTOR (DEP domain containing mTOR-interacting protein), mLST8 (mammalian lethal with sec-13 protein 9), and a Tti1-Tel2 complex [6–8, 10, 12]. Unique to mTORC1 are Raptor (regulatory-associated protein of mTOR) and the inhibitory PRAS40 (proline-rich AKT substrate) subunit which enable it to phosphorylate the downstream ribosomal protein p70S6K (Ser^235/236^) and 4FBP1 (eukaryotic initiation factor 4E-binding protein 1) [14]. Activation of mTORC1 is responsible for promoting autophagy and ribosome biogenesis [12, 15, 16]. mTORC2 contains Rictor (Rapamycin-insensitive companion of mTOR) and mSin1 (mammalian stress-activated MAP kinase-interacting protein), allowing for phosphorylation of downstream AKT^473^ and GSK3ß [17]. Less is known about mTORC2 than its counterpart, but it has been proven to play a key role in cytoskeletal organization and cell growth/survival [18].

In 2006, our laboratory first identified the preconditioning-like cardioprotective effects of Rapamycin against ischemia/reperfusion (I/R) injury [9]. Rapamycin pretreatment reduced infarct size after I/R injury and also attenuated necrosis and apoptosis in cardiomyocytes following simulated ischemia/reoxygenation (SI-RO). Mechanisms mediating the protective effects of Rapamycin involved the opening of mitochondrial ATP-sensitive potassium channels [9]. We further demonstrated that additional cardioprotective signaling occurs via phosphorylation of ERK, STAT3, and endothelial nitric oxide synthase (eNOS), in conjunction with an increased Bcl-2 : Bax ratio [19]. Whether Rapamycin treatment attenuates reperfusion injury remains unknown. Therefore, the present study was designed to investigate the* in vivo* effects of reperfusion therapy with Rapamycin. Considering the important role of the Reperfusion Injury Salvage Kinase (RISK) pathway, a group of prosurvival protein kinases (including AKT and Erk1/2), which confer cardioprotection when activated specifically at the time of myocardial reperfusion [20, 21], we hypothesized that the RISK pathway may provide an amenable pharmacological target for cardioprotection with Rapamycin. Our results demonstrate that Rapamycin administered at the onset of reperfusion shows great promise as an interventional drug capable of significantly reducing infarct size and cardiomyocyte apoptosis following I/R injury via signaling pathways involving MAP kinases and the PI3K-AKT.

## 2. Methods

### 2.1. Chemicals and Reagents

Rapamycin (Sirolimus®) was purchased from LC Laboratories (MA). PD98059, an ERK inhibitor, was purchased from Sigma Aldrich (St. Louis, MO). ApoAlert™ DNA Fragmentation Assay kit was purchased from BD Biosciences, Palo Alto, CA. DAPI was bought from Vector Laboratories, Inc. CA. Antibodies for phospho-serine^473^ AKT, AKT, p-S6, S6, p-ERK1/2, ERK1/2, p-P38, P38, phospho-tyrosine^705^-STAT3, STAT3, Bcl-2, and Bax were purchased from Cell Signaling Technology. SOD-2, ferritin heavy chain, and GAPDH-HRP were purchased from Santa Cruz Biotechnology.

### 2.2. Animals

Adult C57BL/6J male mice (body weight ~30 g) were purchased from Jackson Laboratories. Animal care and experiments were approved by the Institutional Care and Use Committee of Virginia Commonwealth University and were conducted according to the Guide for the Care and Use of Laboratory Animals for Biomedical Research published by the National Institutes of Health (No. 85-23, revised 1996).

### 2.3. Experimental Groups

For* in vivo* regional I/R protocol, four groups were used: (1) DMSO (7.5%), (2) Rapamycin (0.25 mg/kg), (3) Rapamycin + PD98059 (1 mg/kg an ERK inhibitor), and (4) PD98059 alone. DMSO or Rapamycin was injected (intracardiac, i.c.) 5 min before the onset of reperfusion following 30 min of* in situ* ischemia. ERK activation was inhibited through intraperitoneal administration of PD98059 10 minutes following the start of ischemia. The surgeon was blinded to the treatment allocation.

### 2.4. Myocardial Infarction Protocol

**Figure 1:**
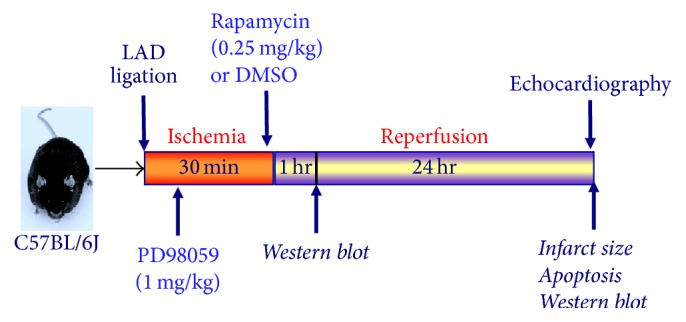
Experimental protocol. Experimental groups and protocol of regional I/R by LAD occlusion in C57 mouse hearts.

The* in vivo* myocardial I/R procedures were conducted in mouse by ligation of the left anterior descending coronary artery (LAD) according to a previously reported method [22]. Animals were anesthetized with pentobarbital sodium (70 mg/kg, i.p.) and ventilated on a positive pressure ventilator. A left thoracotomy was performed at the fourth intercostal space, and the heart was exposed by stripping the pericardium. To induce ischemia, the LAD was occluded for 30 min by a 7.0 silk ligature that was placed around it and a small piece of polyethylene tubing (PE10) that was positioned on top of it. Reperfusion was established by removing the PE10 tube that was compressing the LAD. 5 min prior to reperfusion, Rapamycin (0.25 mg/kg) or DMSO (7.5%) was injected intracardially to ensure its immediate availability at the onset of reperfusion as well as avoiding any preconditioning effect. Although intracardial injection of Rapamycin is not clinically applicable, it resembles the best option to mimic intracoronary delivery during cardiac catheterization, which is not feasible in mice. We chose this route of administration, which has been previously reported in the literature with same myocardial ischemia/reperfusion protocol [23], to bypass liver metabolism that occurs with intravenous/intraperitoneal administration in order to ensure immediate availability of Rapamycin at the onset of reperfusion in our study. The dose of Rapamycin was chosen based on our previous studies on Rapamycin-induced cardioprotection against myocardial* I/R* injury [9, 19]. Air was then expelled from the chest, and the chest cavity was closed. The animal was placed in a cage on a heating pad until fully conscious and then kept for 24 hours (Figure 1).

### 2.5. Measurement of Infarct Size

The heart was removed following 30 min of ischemia and 24 hours of reperfusion and mounted on a Langendorff apparatus. The coronary arteries were perfused with 37°C Krebs-Henseleit buffer. After the blood was washed out, 3 mL of 10% TTC in isotonic phosphate buffer (pH 7.4) at 37°C was infused over several minutes before the ligature was retightened and ~1 mL of 5% Phthalo blue dye was injected as a bolus into the aorta until most of the heart turned blue. The heart was perfused with saline to wash out the excess dye. After freezing the heart for 24 hours, it was sectioned from apex to base into slices of equal thickness (~1 mm). The slices were then fixed in 10% neutral buffered formaldehyde for 4 hours with a weight on top to keep the heart slices flat for the initial 30 min. The areas of infarcted tissue, the risk zone, and the whole left ventricle were determined by computer morphometry using ImageJ imaging software (NIH, Bethesda).

### 2.6. Echocardiography

Cardiac function was assessed via echocardiography using a VisualSonics Vevo 2100 Imaging System. Mice were anesthetized with 2.5 L/min Isoflurane prior to assessment. Imaging was conducted through the parasternal short axis while mice were exposed to 1.5 L/min Isoflurane via a nose cone. Left ventricular (LV) end-diastolic diameter (LVEDD) and end-systolic diameter (LVESD), LV fractional shortening (FS), and ejection fraction (EF) were calculated using Vevo analysis software (version 2.2.3).

### 2.7. Apoptosis Assay

Apoptosis was determined by TUNEL staining using an ApoAlert™ DNA Fragmentation Assay kit (BD Biosciences, CA) according to the manufacturer's protocol. Hearts were stored in a 10% formalin solution, and paraffin embedded tissue section was mounted on glass slides. Apoptosis was then assessed in the transverse sections of paraffin sections as previously reported [19]. Apoptotic cells were examined under a fluorescence microscope (Nikon Eclipse T*i*) which were clearly identified with a strong nuclear green fluorescence. All cell nuclei were visualized as blue fluorescence following staining with DAPI. The apoptotic index was expressed as the number of apoptotic cells of all cardiomyocytes per field. Apoptotic rate in the peri-infarct regions was calculated using 6 random fields.

*Lipid Peroxidation Assay*. Lipid peroxidation in heart was assayed by measuring malondialdehyde (MDA) using a lipid peroxidation assay kit (BioVision, CA, USA) according to the manufacturer's protocol as previously reported [7].

### 2.8. Western Blots

Total soluble protein was extracted from the whole heart tissues (following 30 min ischemia and 1 hr reperfusion or 24 hr reperfusion) with extraction buffer. Seventy-five (75 *μ*g) proteins from each sample were separated by SDS-PAGE and transferred onto nitrocellulose membrane (0.2 um pore size). The membrane was incubated in cold conditions overnight with primary antibodies (phospho-serine^473^-AKT, AKT, phospho-serine^235/236^-S6, S6, p-ERK1/2, ERK1/2, p-P38, P38, phospho-tyrosin^705^-STAT3, STAT3, Bcl-2, Bax, SOD-2, and ferritin heavy chain 1 or GAPDH-HRP) in a 1 : 1000 dilution with 5% BSA. The membrane was washed and incubated with horseradish peroxidase conjugated secondary antibody (1 : 3000 dilution in 5% milk solution) and the blots were developed using a chemiluminescent system (Western Lighting Plus-ECL; Perkin Elmer, Inc.).

### 2.9. Data Analysis and Statistics

Statistical analysis was performed with GraphPad Prism 7 (Graphpad Software Inc.). Data are presented as mean ± SEM. The differences between groups were analyzed with one-way analysis of variance (ANOVA) followed by Student-Newman-Keuls post hoc test for pairwise comparison, or by unpaired *t*-test. *p* < 0.05 was considered to be statistically significant.

## 3. Results

### 3.1. Survival

A total of 105 mice were used in this study; 26 out of 30 mice survived in the Rapamycin-treated group (approximately 86%) as compared with 27 out of 39 in the DMSO-treated group (69%) following I/R. Post-I/R survival rate was 63% (14 out of 22) in mice treated with Rapamycin plus PD98059 and 57% (8 out of 14) in mice treated with PD98059 alone.

### 3.2. Reperfusion Therapy with Rapamycin Reduces Infarct Size and Improves Cardiac Function

**Figure 2:**
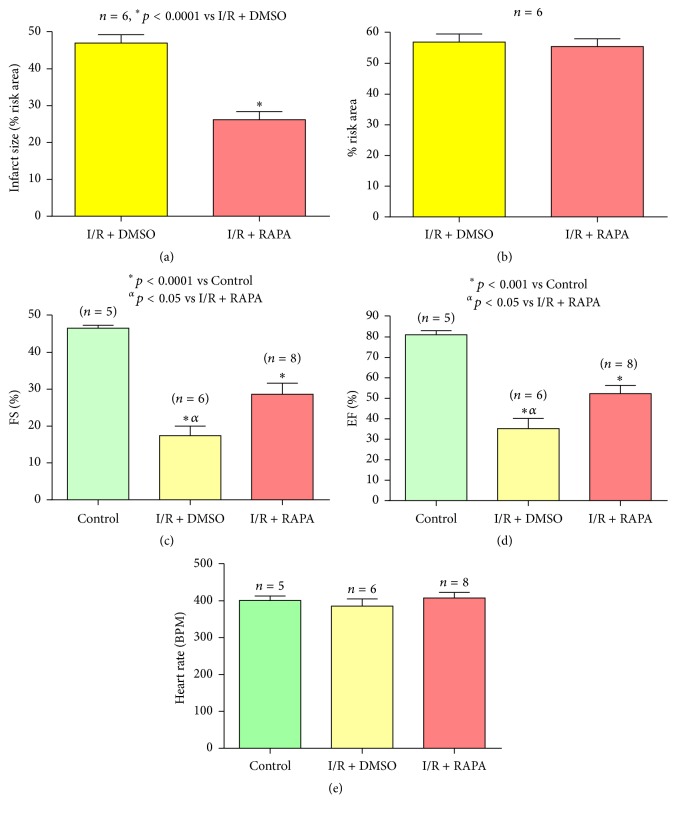
Rapamycin at the onset of reperfusion reduces infarct size. (a) Infarct size of C57 mouse hearts following 30 minutes of ischemia and 24 hours of reperfusion with and without Rapamycin treatment as percentage of risk area. After TTC and Phthalo blue staining, the blue area represents the remote area, the area at risk is stained red, and the infarcted myocardium is stained white. (b) Risk area as percentage of the overall tissue section. (c) Fractional shortening (%) as measured via echocardiography. (d) Ejection fraction (%). (e) Heart rate (BPM).

Rapamycin treatment at the onset of reperfusion resulted in a significant reduction in infarct size (% of risk area) to 26.2 ± 2.2% as compared to 47.0 ± 2.3% in the DMSO-treated Control group (*n* = 6, *p* < 0.0001) after 30 min ischemia and 24-hour reperfusion (Figure 2(a)). Total risk area was not statistically different between Rapamycin (55.5% ± 2.5) and DMSO (56.9% ± 2.6) treated groups (*n* = 6, *p* > 0.05) following* in vivo* I/R (Figure 2(b)).

Cardiac function was assessed by echocardiography following 30 min ischemia by LAD occlusion and 24-hour reperfusion. DMSO-treated mice showed significant reduction of FS, 17.4 ± 2.6%, and EF, 35.2 ± 4.99%, after I/R as compared to Control mice before I/R (FS, 46.6 ± 0.7%, and EF, 81.0 ± 2.1%) (*n* = 6, ^*∗*^*p* < 0.001 versus Control; Figures 2(c) and 2(d)). Rapamycin improved post-I/R FS (28.63 ± 3.01%) and EF (52.22 ± 4.1%). Heart rates did not change between the groups (Figure 2(e)).

### 3.3. Effect of Rapamycin on Myocardial Apoptosis

**Figure 3:**
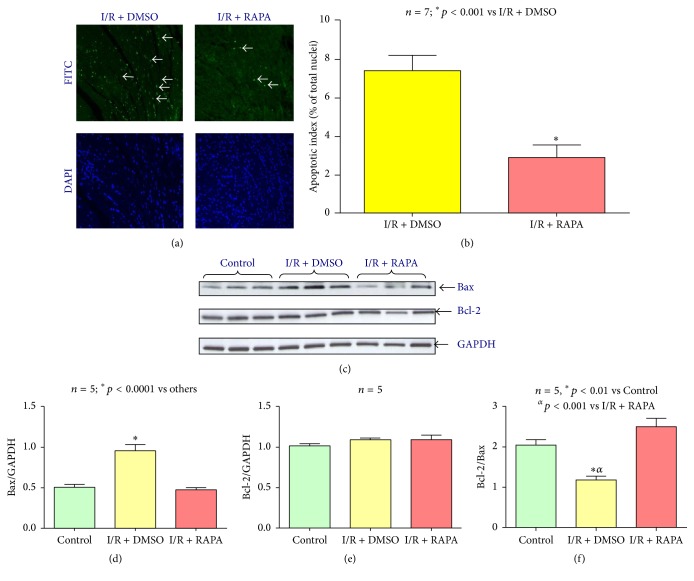
Effect of Rapamycin on myocardial apoptosis. (a) Representative images of TUNEL-positive nuclei in green fluorescent color and total nuclei staining with DAPI (blue) staining of sections of myocardium treated following 30 minutes of ischemia/24 hours of reperfusion. (b) Apoptotic index calculated as the % of TUNEL stained apoptotic nuclei versus the total amount of nuclei visualized via DAPI staining. (c) Representative immunoblots of Bax and Bcl-2 in hearts of C57 mice following 30 minutes of ischemia and 24 hours of reperfusion including Control (C57), DMSO (I/R + DMSO), and Rapamycin (I/R + RAPA) treated groups (*n* = 3/group). GAPDH immunoblot was used as a loading Control. (d) Densitometric analysis of the ratio of Bax to GAPDH (*n* = 5/group). (e) Densitometric analysis of the ratio of Bcl-2 to GAPDH (*n* = 5/group). (f) Densitometric analysis of the ratio of Bcl-2 to Bax (*n* = 5/group).

Following* in vivo* I/R, tissue sections from Rapamycin-treated hearts showed a significant reduction in TUNEL-positive nuclei in the peri-infarct regions (2.87 ± 0.64%) as compared to the DMSO-treated group (7.39 ± 0.81%, *n* = 7, *p* < 0.001) (Figures 3(a) and 3(b)).

### 3.4. Rapamycin Blocks Proapoptotic Bax Signaling

The expressions of Bcl-2 and Bax were measured to identify prosurvival and proapoptotic cell signaling, respectively. Bax expression was significantly increased at 24 hours following* in vivo* I/R (*n* = 5, *p* < 0.0001) but was reduced to a preischemic state with administration of Rapamycin at reperfusion (*n* = 5, *p* < 0.0001) (Figures 3(c) and 3(d)). The expression of Bcl-2 did not change following I/R injury with/without Rapamycin treatment (Figures 3(c) and 3(e)). However, the ratio of Bcl-2/Bax significantly declined following I/R (*n* = 5, *p* < 0.01) and was restored by Rapamycin treatment (Figure 3(f)).

### 3.5. Rapamycin Inhibits mTORC1 and Promotes mTORC2 Activity

**Figure 4:**
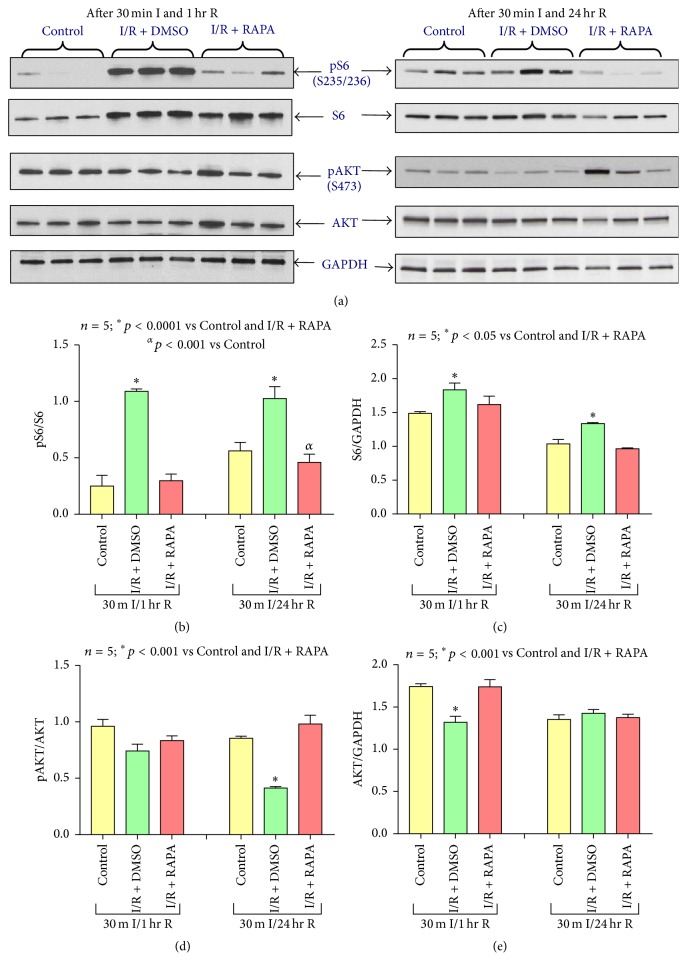
Rapamycin inhibits mTORC1 while simultaneously promoting mTORC2 activity. (a) Representative immunoblots of phosphorylated S6 (S235/236), total S6, phosphorylated AKT (S473), and total AKT in hearts of C57 mice following 30 minutes of ischemia and 1 hour and 24 hours of reperfusion including Control (C57), I/R + DMSO, and Rapamycin (I/R + RAPA) treated groups (*n* = 3/group). GAPDH immunoblots were used as a loading Control. (b) Densitometric analysis of the ratio of pS6 to S6. (c) Densitometric analysis of the ratio of S6 to GAPDH (*n* = 5/group). (d) Densitometric analysis of the ratio of pAKT to AKT (*n* = 5/group). (e) Densitometric analysis of the ratio of AKT to GAPDH (*n* = 5/group).

To further elucidate the effects of Rapamycin on mTOR, S6 and Akt phosphorylation levels were analyzed by Western blot to gauge mTORC1 and mTORC2 activity (Figure 4(a)), respectively. We performed Western blots using the proteins isolated from mouse heart following 30 min of ischemia and 1 hour or 24 hours of reperfusion. S6 phosphorylation at Ser 235/236 was immensely increased following ischemia and 1 hr as well as 24 hours of reperfusion as compared to Control (Figures 4(a) and 4(b)). The phosphorylation of S6 was significantly reduced with Rapamycin treatment (*n* = 5, *p* < 0.0001) (Figures 4(a) and 4(b)). Total S6 was also induced following I/R as compared to Control, but it significantly reduced with Rapamycin treatment as compared to Control or I/R (*n* = 5, *p* < 0.05) (Figures 4(a) and 4(c)). The phosphorylation of AKT (Ser^473^) was reduced following ischemia and 24 hours of reperfusion as compared to Control, although that reduction was not significant after 1 hour of reperfusion (Figures 4(a) and 4(d)). However, postischemic Rapamycin treatment was found to significantly increase phosphorylation at this site following 30 min ischemia and 24-hour reperfusion (*n* = 5, *p* < 0.001) (Figures 4(a) and 4(d)). The induction of phosphorylation of AKT following 1 hour of reperfusion with Rapamycin was not significant. Total AKT levels were reduced following 30 min ischemia and 1-hour reperfusion (*n* = 5, *p* < 0.001) but were not altered between groups following 24 hours of reperfusion (Figures 4(a) and 4(e)).

### 3.6. Effect of Rapamycin on MAP Kinase Signal Transduction

**Figure 5:**
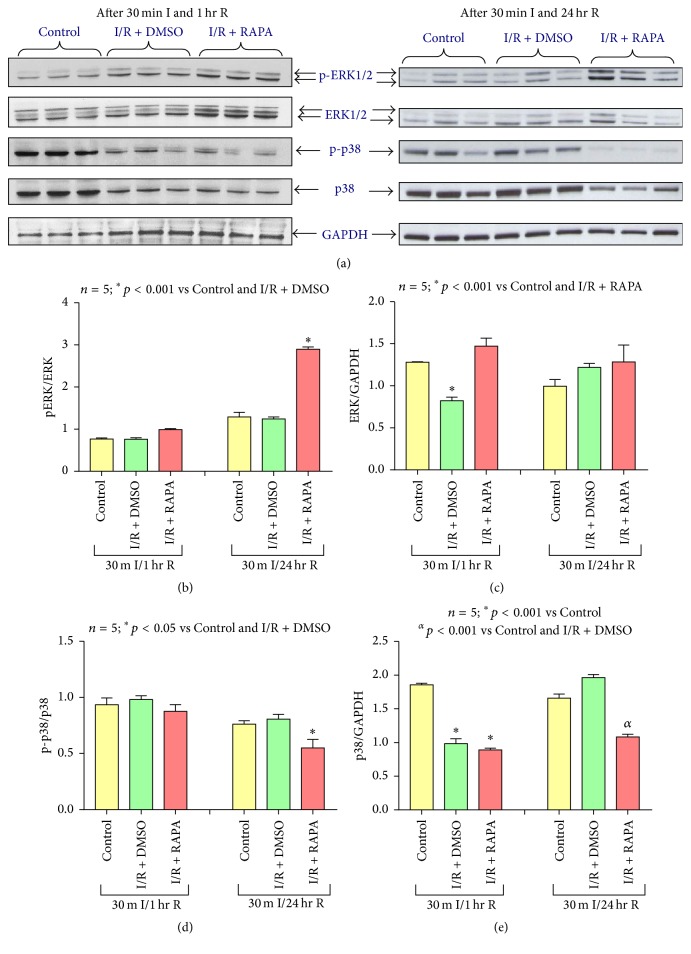
Effect of Rapamycin on MAP kinase signaling. (a) Representative immunoblots of phosphorylated ERK1/2, total ERK1/2, phosphorylated P38, and total P38 in hearts of C57 mice following 30 minutes of ischemia and 1 hour and 24 hours of reperfusion including Control (C57), DMSO (I/R + DMSO), and Rapamycin (I/R + RAPA) treated groups (*n* = 3/group). GAPDH immunoblots were used as a loading Control. (b) Densitometric analysis of the ratio of pERK1/2 to ERK1/2 (*n* = 5/group). (c) Densitometric analysis of the ratio of ERK1/2 to GAPDH (*n* = 5/group). (d) Densitometric analysis of the ratio of pP38 to P38. (e) Densitometric analysis of the ratio of P38 to GAPDH (*n* = 5/group).

To analyze the effect of postischemic Rapamycin treatment on the MAP kinase signaling pathways following 1 hour or 24 hours of reperfusion, phosphorylation levels of ERK1/2 and P38 were measured via Western blot analysis (Figure 5(a)). p-ERK1/2 was not altered following 1 hour of reperfusion between groups. However, following 24 hours of reperfusion, p-ERK1/2 was significantly increased following postischemic treatment with Rapamycin as compared to the Control and DMSO-treated I/R groups (*n* = 5, *p* < 0.001) (Figures 5(a) and 5(b)). Total ERK1/2 level significantly reduced following 1 hour of reperfusion (*n* = 5, *p* < 0.001) but it was rescued by Rapamycin treatment. However, total ERK did not change among any of the treatment groups following 24 hours of reperfusion (*n* = 5, *p* > 0.05) (Figures 5(a) and 5(c)). Phospho-p38 remained unchanged between Control and DMSO-treated I/R groups (*n* = 5, *p* > 0.05) following 1 hour and 24 hours of reperfusion, but it significantly decreased with Rapamycin treatment following 24 hours of reperfusion (*n* = 5, *p* < 0.01) (Figures 5(a), 5(d), and 5(e)).

### 3.7. ERK Inhibition Abolishes the Infarct Sparing Effect of Rapamycin

**Figure 6:**
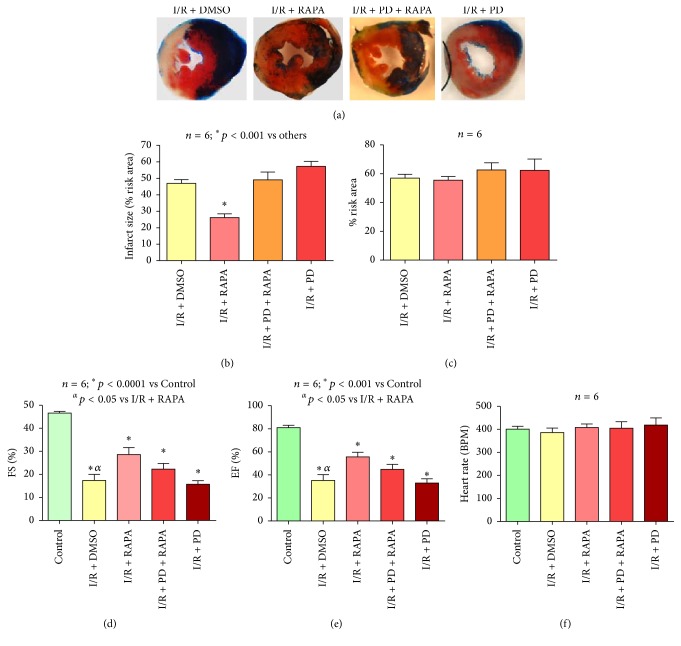
Effect of ERK inhibition on the infarct sparing effects of Rapamycin. (a) Whole tissue TTC and Phthalo blue staining of C57 mouse hearts following 30 minutes of ischemia and 24 hours of reperfusion with Rapamycin and/or PD98059. (b) Infarct size as a percentage of risk area. (c) Risk area as percent of LV. (d) Fractional shortening (%) as measured by echocardiography. (e) Ejection fraction (%). (f) Heart rate (BPM).

Rapamycin treatment significantly reduced infarct size (to 26.2 ± 2.2% from 47.0 ± 2.3% in the DMSO-treated Control group), which was completely blocked by treatment with ERK inhibitor, PD98059 (49.1 ± 4.7%, *n* = 6, *p* < 0.0001 versus I/R + RAPA, Figure 6(b)). PD98059 alone did not alter the infarct size compared to DMSO-treated I/R group (57.27 ± 3.0%). Administration of PD98059 with/without Rapamycin did not alter total risk area (62.65% ± 1.8% and 62.36 ± 3.3%) compared to Rapamycin- (55.52% ± 2.5%) and DMSO- (56.98% ± 2.6%) treated I/R groups (*n* = 6, *p* > 0.05) (Figure 6(c)).

Treatment with PD98059 also abolished post-I/R cardiac functional improvement induced by Rapamycin (FS, 22.31 ± 2.5%, and EF, 44.81 ± 4.4%, *n* = 6, Figures 6(d) and 6(e)). Post-I/R cardiac function was not significantly different between PD98059 and DMSO-treated I/R mice (FS, 15.73 ± 1.5%, and EF, 32.95 ± 3.6%). Heart rate did not change between the groups (Figure 6(f)).

**Figure 7:**
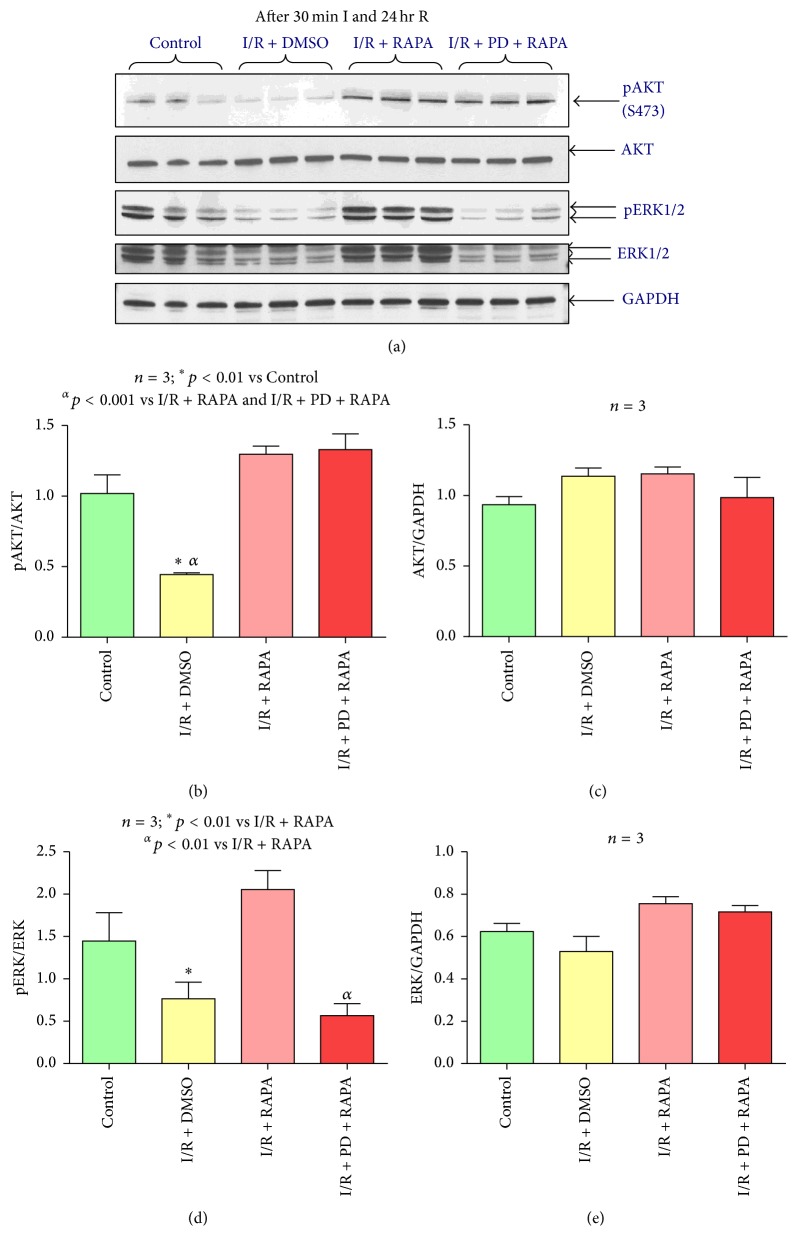
Effect of ERK inhibitor on phosphorylation of AKT and ERK. (a) Representative immunoblots of phosphorylated AKT, total AKT, phosphorylated ERK1/2, total ERK1/2, and GAPDH in hearts of C57 mice following 30 minutes of ischemia and 24 hours of reperfusion including Control (C57), DMSO (I/R + DMSO), Rapamycin (I/R + RAPA), and PD (I/R + PD + RAPA) treated groups (*n* = 3/group). (b) Densitometric analysis of the ratio of pAKT/AKT, (c) AKT/GAPDH, (d) pERK1/2 to ERK1/2, and (e) ERK1/2 to GAPDH.

To investigate the effect of PD98059 on myocardial activation of ERK and AKT, we performed Western blot analysis using myocardial proteins from mice subjected to 30 min ischemia and 24-hour reperfusion and treated with PD98059 and Rapamycin. The results show that PD98059 inhibited Rapamycin-induced phosphorylation of ERK (Figures 7(a) and 7(d)) but had no effect on the induction of phosphorylation of AKT by Rapamycin (Figures 7(a) and 7(b)).

### 3.8. Rapamycin Attenuates Lipid Peroxidation through ERK Activation

**Figure 8:**
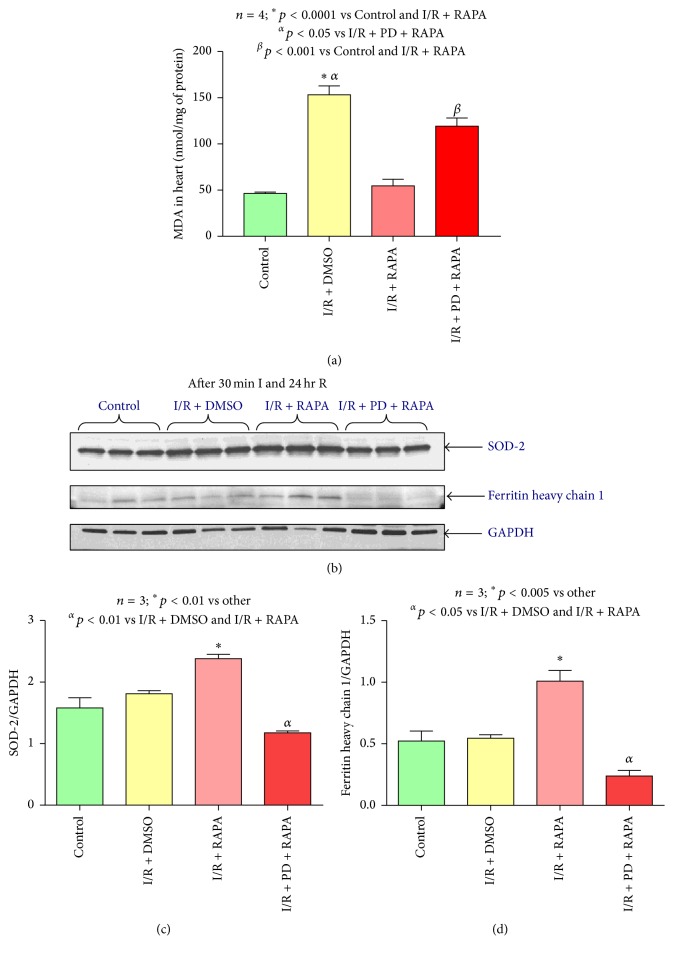
ERK inhibition abolishes the antioxidant effect of Rapamycin. (a) MDA level in hearts of C57 mice following 30 min ischemia and 24 hours of reperfusion with/without Rapamycin (RAPA) and/or PD98059 (RAPA + PD). (b) Representative immunoblots of SOD-2, ferritin heavy chain 1, and GAPDH in hearts of C57 mice following 30 minutes of ischemia and 24 hours of reperfusion including Control (C57), DMSO (I/R + DMSO), and Rapamycin (I/R + RAPA) and PD98059 (I/R + PD + RAPA) treated groups (*n* = 3/group). (c) Densitometric analysis of the ratio of SOD-2 to GAPDH (*n* = 3/group). (d) Densitometric analysis of the ratio of ferritin heavy chain to GAPDH (*n* = 3/group).

The oxidative stress as measured by accumulation of lipid peroxidation product malondialdehyde (MDA) was significantly increased in the hearts after 24-hour I/R injury as compared to nonischemic Control hearts (*n* = 4; *p* < 0.0001, Figure 8(a)). Rapamycin treatment at the onset of reperfusion significantly attenuated MDA accumulation following I/R (*n* = 4, *p* < 0.0001), suggesting a potent antioxidant-type activity of Rapamycin. Interestingly, ERK inhibitor PD98059 blocked the antioxidant effect of Rapamycin (*n* = 4, *p* < 0.001). Postischemic treatment with Rapamycin also induced the expression of antioxidant enzymes (SOD-2, manganese-dependent superoxide dismutase, and ferritin heavy chain 1) in hearts as compared to Control and DMSO-treated I/R groups (Figures 8(b), 8(c), and 8(d)). Again, PD98059 abolished the induction of both antioxidant proteins with Rapamycin.

### 3.9. Effect of Rapamycin on STAT3 Signaling

**Figure 9:**
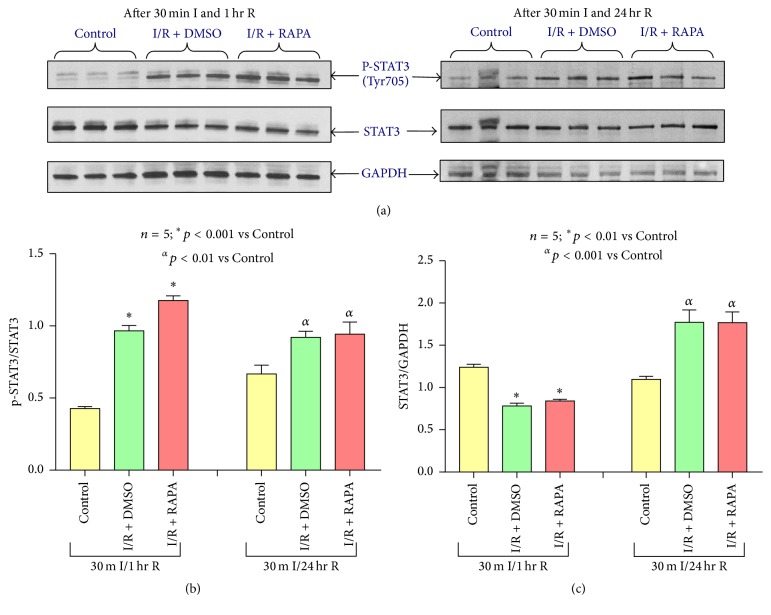
Effect of Rapamycin on STAT3 signaling. (a) Representative immunoblots of phosphorylated STAT3 and total STAT3 in hearts of C57 mice following 30 minutes of ischemia and 1 hour and 24 hours of reperfusion including Control (C57), DMSO (I/R + DMSO), and Rapamycin (I/R + RAPA) treated groups (*n* = 3/group). GAPDH immunoblots were used as a loading Control. (b) Densitometric analysis of the ratio of pSTAT3 to STAT3 (*n* = 5/group). (c) Densitometric analysis of the ratio of STAT3 to GAPDH (*n* = 5/group).

Phosphorylation of STAT3 was significantly induced following I/R (*n* = 5, *p* < 0.001 after 1 hour of reperfusion and *p* < 0.01 after 24 hours of reperfusion versus Control) (Figures 9(a) and 9(b)). Rapamycin treatment at reperfusion could not alter postischemic induction of phosphorylation of STAT3. Total STAT3 significantly reduced following ischemia and 1 hour of reperfusion (*n* = 5, *p* < 0.01 versus control), but it was induced after 24 hours of reperfusion (*n* = 5; *p* < 0.001 versus Control) (Figures 9(a) and 9(c)). Rapamycin could not also alter the postischemic STAT3 levels following 1 hour as well as 24 hours of reperfusion.

## 4. Discussion

Early restoration of coronary flow is obligatory to resuscitate the ischemic myocardium. However, reperfusion may paradoxically contribute to myocardial dysfunction in the ischemic area via “reperfusion injury” [24–26]. Reperfusion injury results in cardiomyocyte damage through ventricular arrhythmias, myocardial stunning, microvascular obstruction, endothelial dysfunction, and irreversible cell damage or necrosis (termed lethal reperfusion injury) [27, 28]. As it stands, mitigation of reperfusion-induced cardiomyocyte death remains among the most attractive approaches to further improve outcomes following successful percutaneous coronary intervention in AMI patients. However, no pharmaceutical approaches are currently approved by the FDA that can directly protect heart against the deleterious effects of reperfusion injury due to the lack of a consistent clinical benefit.

In the present study, we investigated the potential therapeutic effect of Rapamycin when given at reperfusion in a mouse model of* in vivo* I/R. Our results show that Rapamycin (0.25 mg/kg, i.c.) induced cardioprotection as shown by significant reduction of myocardial infarct size. In addition post-I/R cardiomyocyte apoptosis was significantly diminished in the Rapamycin-treated group as compared to DMSO-treated mice as depicted by TUNEL staining. Echocardiography revealed that cardiac function was preserved in Rapamycin-treated mice as compared to Control mice at 24 hours following I/R. Moreover, Rapamycin treatment inhibited the expression of proapoptotic protein Bax following I/R, without alteration of antiapoptotic protein Bcl-2. The ratio of Bcl-2/Bax expression was significantly increased in Rapamycin-treated group suggesting a potent antiapoptotic effect in the myocardium following I/R.

Rapamycin selectively binds to FKBP12 and inhibits the kinase activity of mTORC1 [4, 6]. When bound, FKBP12 restricts access of mTOR substrate docking sites and destabilizes the structural integrity of mTORC1 [12, 29–31]. In acute doses, such as those utilized in the present study, mTORC2 is unaffected by this drug-receptor complex [18, 32]. mTOR, being a major growth regulator, plays an integral role in the induction of cardiac hypertrophy in response to pressure overload and MI [33]. Selective inhibition of mTORC1 with Rapamycin has been shown to preserve cardiac function and attenuate maladaptive hypertrophy resulting from these stressors [34–37].

Recent studies demonstrated that mTORC1 is the dominant complex stimulated by I/R injury, with mTORC2 showing no significant changes in activity [38]. Genetic and pharmacologic evidence shows that mTORC1 inhibition is beneficial after myocardial infarction [39–41]; however, genetic deletion of mTORC2 is associated with increased ischemic damage [42]. PRAS40 overexpression reduced mortality and improved cardiac function after myocardial infarction via increased activity of mTORC2 signaling [42]. In the present study, we used the phosphorylation of S6 (Ser^235/236^) and AKT (Ser^473^) as measures for mTORC1 and mTORC2 activity, respectively. We examined the phosphorylation levels of S6 and AKT following 30 min of ischemia and 1 hour (early effect of reperfusion) as well as 24 hours of reperfusion (late effect of reperfusion).

Our results show that the phospho-S6 level increased following I/R, and the induction of phospho-S6 was abolished following treatment with postischemic Rapamycin therapy after 1 hour and 24 hours of reperfusion. Phospho-AKT levels showed no remarkable changes following I/R, but Rapamycin administration induced the phosphorylation of AKT after 24 hours of reperfusion. These results suggest that treatment with Rapamycin at reperfusion inhibits mTORC1, while simultaneously promoting mTORC2 signaling. This upregulation of phospho-AKT (Ser^473^) in Rapamycin-treated mice at reperfusion may confer myocardial protection against I/R injury, which is comparable to that afforded by ischemic preconditioning [41].

The RISK pathway, representing a group of survival protein kinases that include the PI3K/AKT and ERK1/2, has been reported to exert cardioprotection when activated specifically at the time of myocardial reperfusion [20]. The cardioprotective effect of RISK pathway activation is associated with the recruitment of antiapoptotic signaling and the inhibition of proapoptotic proteins by avoiding the opening of the mitochondrial permeability transition pore at the onset of reperfusion [43–45]. Several studies have demonstrated that activating the RISK pathway with pharmacological agents may be a viable intervention for limiting myocardial infarction. Specifically, insulin, growth factors, TGB-*β*1, leptin, apelin, opioids, adenosine, bradykinin, atorvastatin, metformin, guanylyl cyclase receptor atrial natriuretic peptide, and glucagon-like peptide elicit cardioprotection at the time of myocardial reperfusion through the activation of the RISK pathway [44]. In the present study, treatment with Rapamycin at the onset of reperfusion specifically activated AKT by inducing phosphorylation at the Ser 473 residue which may provide cardioprotective benefits.

The MAPK family of proteins encompass the second arm of the RISK signal pathway. While the apoptosis-inhibiting and infarct-reducing benefits of ERK1/2 upregulation have been well documented across multiple treatment models, there is still great debate regarding the role of p38 in cardioprotection [46–59]. In the present study, a significant increase in ERK1/2 phosphorylation was observed following Rapamycin administration at the onset of reperfusion and following 24 hours of reperfusion. Additionally, hearts subjected to I/R without any pre- or posttreatment elicited no change in total ERK following 24 hours of reperfusion. These results are in line with previous studies published by our laboratory demonstrating the beneficial effects of preconditioning with Rapamycin as well as sildenafil (a phosphodiesterase inhibitor) [19, 49]. p38 is particularly unique in the temporal aspects of its activation and inhibition. Despite being activated by myocardial I/R, previous evidence showed that preischemic inhibition of P38 MAPK reduced infarct size and preserved cardiac function in both* in vivo* and* ex vivo* mouse, rat, rabbit, and pig models [19, 49, 60–66]. A more recent study demonstrated that the benefits of p38 inhibition are time sensitive, with infarct-induced arrhythmias being attenuated only when p38 was inhibited before or during ischemia [67]. Conversely, ischemic preconditioning (IPC) has been shown to promote p38 phosphorylation at the time of myocardial reperfusion, indicating that modulation of these MAP kinases is subject to a complex array of signal transduction pathways over the entire course of ischemia and reperfusion [47, 50, 52]. In the present study, phosphorylation of p38 was not altered after 1 hour of reperfusion between groups, but it has been inhibited following 24 hours of reperfusion with Rapamycin treatment. Total p38 was inhibited after ischemia and 1 hour of reperfusion, but it was induced following 24 hours of reperfusion. Rapamycin reduced the total p38 after 24 hours of reperfusion.

Inhibition of ERK via administration of the ERK inhibitor, PD98059, was performed in order to understand the cause and effect relationship between ERK activation and the reduction of infarct size following Rapamycin treatment at the end of ischemia. PD98059 completely abolished Rapamycin-induced infarct size reduction which was comparable with the DMSO group. Rapamycin-induced cardiac functional improvement following I/R was also abolished following treatment with PD98059. These results suggest that ERK activation is a key factor in the cardioprotective benefits of Rapamycin therapy and is responsible for its overall infarct size reduction. PD98059 inhibited Rapamycin-induced ERK phosphorylation following 24 hours of reperfusion, which was not significantly different as compared to I/R group. However PD98059 had no effect on Rapamycin-induced AKT phosphorylation. These results suggest that Rapamycin-induced AKT phosphorylation may have triggered ERK phosphorylation, because pharmacological inhibition of ERK had no effect on phosphorylation of AKT. Future studies are required to identify the precise sequence of activation of RISK signaling.

Many studies showed that reactive oxygen species (ROS) generated during early reperfusion lead to extensive oxidative stress to cells, which contributes to irreversible ischemic myocardial injury [24, 26, 68]. Earlier studies demonstrated the antioxidant effect of Rapamycin by protecting mitochondria against oxidative stress and apoptosis in a rat model of Parkinson disease [69], by improving endothelial function in aging [70] and vascular contractility [71]. Rapamycin also protects human corneal endothelial cells from oxidative injury-mediated cell death via inhibition of ROS production [72]. We previously reported the antioxidant effect of Rapamycin in reducing diabetes-induced myocardial dysfunction [7]. Rapamycin treatment reduced oxidative stress in diabetic heart by augmenting antioxidant proteins as well as iron-regulating proteins [7]. Ferritin heavy chain (FHC) protein plays an important role in the pathogenesis of heart failure [73]. A decrease in the abundance of ferritin heavy chain 1 enhances the levels of iron deposition and the resultant enhanced oxidative stress, which lead to cardiomyocytes cell death. In the present study, our results show that the lipid peroxidation product MDA was enhanced following I/R in the heart, which was reduced by Rapamycin administration at the onset of reperfusion. The expressions of antioxidant proteins SOD-2 and iron-regulating protein ferritin heavy chain 1 were increased with Rapamycin treatment at the onset of reperfusion. Taken together, these data suggest that Rapamycin inhibits oxidative stress by inducing the expression of antioxidant enzyme as well as iron-regulating protein following I/R injury.

In an effort to investigate the effects of Rapamycin as a clinical intervention for MI, we have collected strong evidence detailing the molecular signaling pathways by which the drug acts when administered at the onset of reperfusion. In addition to mTORC1 inhibition and mTORC2 induction, our data proves that Rapamycin also upregulates the ERK protein of the MAPK family. Recently we showed the beneficial effects of chronic treatment with Rapamycin in improving metabolic status and preventing cardiac dysfunction in diabetic mice (db/db mice) through attenuation of oxidative stress as well as alteration of contractile and glucose metabolic proteins [6, 7, 13]. Using diabetic mice, we demonstrated that reperfusion therapy with Rapamycin in diabetic hearts provides cardioprotection through the STAT3-AKT signaling pathway [6, 7, 13]. In the present study, we examined the effect of Rapamycin treatment at the onset of reperfusion on the phosphorylation of STAT3 in nondiabetic and normal mice. The phosphorylation of STAT3 was induced after ischemia and 1 hour as well as 24 hours of reperfusion. Total STAT3 reduced at an early time point of reperfusion but was induced after 24 hours of reperfusion. Rapamycin did not alter the effects of I/R on phosphorylation as well as total level of STAT3 in the hearts of C57 mice. Therefore, the mechanism of cardioprotection against I/R injury by reperfusion therapy with Rapamycin is different in normal mice than in mice with diabetes.

## 5. Conclusion

The novel observation of this present study is that the reperfusion therapy with Rapamycin protects the heart against I/R injury via the AKT and ERK signaling pathway, but not through STAT3 signaling. Because sirolimus-eluting stents are safely used in patients with coronary artery disease, the infarct-reducing and antiapoptotic properties of the drug make it an excellent candidate for therapeutic intervention following an ischemic episode. Future preclinical studies of Rapamycin in higher organisms, including canines and nonhuman primates, would further enhance our understanding of the beneficial effect of reperfusion therapy with Rapamycin [74]. However, the broad-spectrum clinical use of mTOR inhibition is still awaiting a targeted pharmaceutical approach to specifically inhibit mTORC1 with concomitant activation of mTORC2 and the RISK pathway.
